# Perfection

**Published:** 1890-02-22

**Authors:** 


### Words of Consolation.

### PERFECTION.

*For Reading to the Sick.*

Nothing gives so much satisfaction to the mind as the sight of an object perfect in all its parts. A piece of clever machinery doing its work easily and well, a beautiful picture, a flower—for the smallest and poorest herb is a wonder in itself—all alike give more or less pleasure. The two former as the work of man show on a minute inspection, trifling flaws and specks. But God's work is perfection. Examine a rose for instance, the longer we look at it, the more wonderful it seems. The shape and colour first attract the eye, then the rich perfume delights the sense of smell. We examine it more closely, and the lovely texture of the leaves with their graceful curves and exquisite arrangement join with its other merits in making it "A thing of beauty, a joy for ever." Bring a microscope to bear upon it and instead of faults appearing, new charms are discovered every minute, and fill the mind with awe and admiration for the Being Who can by His Word make such marvels.

Perfection and wonders of all sorts are going on around us. The husbandman puts a little seed into the ground and straightway it begins to shoot, the tender leaf comes first, then the ear, afterwards the full corn in the ear. They grow with man's help, till a rich harvest is gained. Man sows and reaps, but God gives the increase. Without His Gift of Life the seed would be barren; without His genial showers the young shoot would wither and die; without His strengthening power it would fall before the cutting blast. So it is with man. God gives him the power of becoming perfect. The seed of virtue is planted in his heart, there to take root, and the dew of the Holy Spirit causes it to grow and flourish. It shoots up into fair promises for the future and then comes the hour of trial. Will they wither and fall off in the heat of the world's temptations, or will they grow and flourish under the cheering beams of the Sun of Righteousness? In this Christian land almost all have been made God's children by Baptism, and the seed of Truth has been sown in their hearts. God says to us as to Abraham of old "walk with Me and be thou perfect." Not perfect in our own strength—that would not be possible—but in Him and the Power of His Might, which He gives us in the Blessed Sacrament of His Love. The sight of sin is odious to God. He hates it because it spoils His Image in which every child of His was made.

Are you a backslider, or careless—you who are now reading these words? Make an earnest determination that you will renounce the devil and all his works and then ask God to give you His Holy Spirit to help you to keep the resolution. Or do you, alas! love wickedness for its own sake because you have broken loose from every law human or divine? Stop! Remember where it will lead you; to destruction and death of body and soul. You were once an innocent child, pure and perfect in God's sight, He loved you then, He loves you now, it is only your sins which are loathsome to Him; He gave Himself for you, He died on the cross as Man for your salvation; He bought you back from the evil one into whose clutches you had fallen and who triumphs in your ruin. Fall on your knees in penitence with a heart softened by the mercies and gifts of our dear Lord, and ask to be forgiven.

